# Direct observation of localized surface plasmon field enhancement by Kelvin probe force microscopy

**DOI:** 10.1038/lsa.2017.38

**Published:** 2017-08-25

**Authors:** Da-Bing Li, Xiao-Juan Sun, Yu-Ping Jia, Mark I Stockman, Hari P Paudel, Hang Song, Hong Jiang, Zhi-Ming Li

**Affiliations:** 1 State Key Laboratory of Luminescence and Applications, Changchun Institute of Optics, Fine Mechanics and Physics, Chinese Academy of Sciences, Changchun 130033, China; 2 Center for Nano-Optics (CeNO) and Department of Physics and Astronomy, Georgia State University, Atlanta, GA 30340, USA

**Keywords:** detector, GaN, KPFM, plasmon

## Abstract

A surface plasmon (SP) is a fundamental excitation state that exists in metal nanostructures. Over the past several years, the performance of optoelectronic devices has been improved greatly via the SP enhancement effect. In our previous work, the responsivity of GaN ultraviolet detectors was increased by over 30 times when using Ag nanoparticles. However, the physics of the SP enhancement effect has not been established definitely because of the lack of experimental evidence. To reveal the physical origin of this enhancement, Kelvin probe force microscopy (KPFM) was used to observe the SP-induced surface potential reduction in the vicinity of Ag nanoparticles on a GaN epilayer. Under ultraviolet illumination, the localized field enhancement induced by the SP forces the photogenerated electrons to drift close to the Ag nanoparticles, leading to a reduction of the surface potential around the Ag nanoparticles on the GaN epilayer. For an isolated Ag nanoparticle with a diameter of ~200 nm, the distribution of the SP localized field is located within 60 nm of the boundary of the Ag nanoparticle. For a dimer of Ag nanoparticles, the localized field enhancement between the nanoparticles was the strongest. The results presented here provide direct experimental proof of the localized field enhancement. These results not only explain the high performance of GaN detectors observed with the use of Ag nanoparticles but also reveal the physical mechanism of SP enhancement in optoelectronic devices, which will help us further understand and improve the performance of SP-based optoelectronic devices in the future.

## Introduction

A surface plasmon (SP) is an optical phenomen on that involves collective oscillations of charges that are confined to nanostructured metal systems^1^. One of the most fundamental properties of a SP is the localization of the electromagnetic field. By nature, the SP eigenmodes of small nanoplasmonic systems are localized and nonpropagating. SP has delivered a number of important applications: ultrasensing^2^, scanning near-field optical microscopy^3^, thermally assisted magnetic recording^4^, biomedical tests^5^ and SP-assisted thermal cancer treatment^6^. Surface plasmon amplification by stimulated emission of radiation (SPASER), a nanoplasmonic counterpart of the laser, was proposed in theory by Bergman and Stockman and has been realized in experiments^7, 8, 9^. Moreover, SP has offered new opportunities to improve the performance of semiconductor optoelectronic devices. Metallic nanostructures have been used to modify the local density of optical states to control light emission, enhance photoluminescence and reduce the quantum-confined Stark effect in light emitting diodes (LEDs)^10, 11, 12^. Plasmonic metallic nanoparticles have also been integrated into photovoltaic devices to increase the solar cell absorption via light-trapping effects and enhance the short-circuit photocurrent density^13, 14, 15, 16, 17^.

In our previous work, we realized high-performance GaN detectors by fabricating Ag nanoparticles on the surface of the GaN epilayer; the responsivity of the GaN detectors increased by over 30 times. The localized SP effect was considered the reason for this enhancement^18, 19^, as elaborated in our previous report. Here, the ‘enhancement’ refers to an increase in responsivity of the treated GaN detector and not to an optical electric field intensity increase due to local plasmonic resonance. However, the direct evidence for this hypothesis is still lacking, and the physical origin behind the SP enhancing the performance of semiconductor photoelectric devices remains to be clarified. Hence, a feasible method is urgently required to explain the SP enhancement.

Here we propose a method to detect the SP-induced localized field variation of Ag nanoparticles on GaN epilayer using Kelvin probe force microscopy (KPFM). As an electrical analog of atomic force microscopy (AFM), KPFM is typically used to observe the surface potential^20, 21, 22, 23^. The electrical properties, such as the polarity^24, 25^, doping type^26, 27^, homogeneity^28^ and charge density^29^ of the sample, have all been identified using KPFM. Based on the theory that the localized field enhancement by SP will adjust the dispersion of the electrons in the GaN epilayer, KPFM was adopted here to verify the existence of the plasmon-enhanced field. Under ultraviolet (UV) illumination, the surface potential around a Ag nanoparticle on the GaN epilayer was reduced, thereby providing direct evidence for the SP-induced localized field enhancement. Our direct observation of the SP effect can open ample avenues to integrate SP into future optoelectronic devices.

## Materials and methods

The undoped GaN epilayer was grown on a (0001) sapphire substrate using metal-organic chemical vapor deposition at 1050 °C. Details of the GaN epilayer growth can be found in previous work^18^. The Ag nanoparticle was fabricated by electron beam evaporation at a pressure of ~5.4 × 10^−6^ mbar. The evaporation rate was ~0.1 nm s^−1^. Next, the sample was annealed at 800 °C for 5 min to form Ag nanoparticles. The Bruker multimode-8 AFM tapping mode was used to characterize the morphology, and the KPFM mode was used to determine the surface potential of the Ag nanoparticles on the GaN epilayer. The tip was a Pt/Ir-coated tip, and the lift height for the interleaved mode was set to be 100 nm. When we used the KPFM to measure the surface potential of the Ag nanoparticles on the GaN epilayer, the laser beam illuminating the tip was reflected on the photodetector. The alternating current (AC) bias was used as an oscillator signal to allow the tip to oscillate at its resonance frequency. The direct current (DC) bias was used to adjust the amplitude of the oscillation to zero as the lock-in signal. In addition, an external UV source was used to enable the measurement of the change of the surface potential without and with UV illumination. Time-domain and frequency-domain finite-element methods were used to simulate the distribution of electric field of a Ag nanoparticle under UV illumination.

## Results and discussion

**Figure 1:**
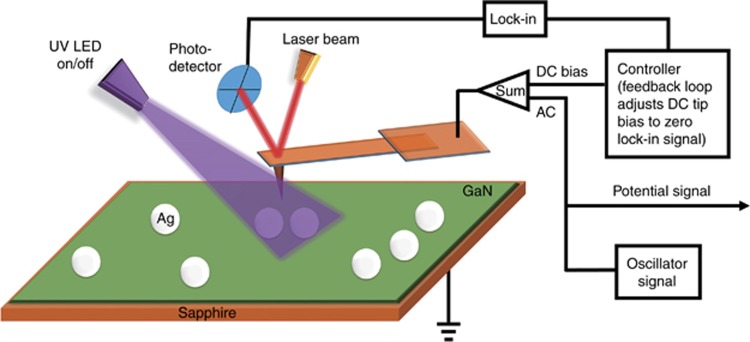
Schematic principle of KPFM. The Ag nanoparticles on the GaN substrate were characterized using KPFM. The laser beam illuminating the tip is reflected to the photodetector, and the amplitude signal is detected. The AC bias is an oscillator signal used to allow the tip to oscillate at its resonance frequency. The DC bias is used to adjust the amplitude of the oscillation to zero as the lock-in signal. In addition, an external UV LED is used to detect the difference in surface potential without and with UV illumination.

A schematic principle of using KPFM to detect the surface potential is given in Figure 1. In KPFM mode, the tip is driven by the applied AC at the same frequency as the resonance frequency (*ω*) of the tip. As the tip scans the sample, it also vibrates. When KPFM is used to measure the surface potential of the Ag nanoparticles on a GaN epilayer, the tip lowers such that it is close to the surface of the sample. At this stage, the contact potential (*V*_CPD_) appears and modulates the vibration of the tip. Moreover, there is a direct voltage biased on the tip (*V*_bias_). The values of *V*_CPD_ and *V*_bias_ are used to determine Δ*V*_DC_ according to Equation (1).


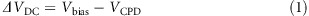


Thus, the active forces on the tip originate from both the alternating voltage (*V*_AC_sin(*ωt*)) and the directive voltage (Δ*V*_DC_)^30, 31, 32^. The equation for the force is given as follows:


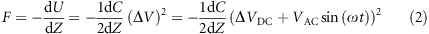


In succession, Equation (3) can be deduced, where the coefficient of Sin(*ωt*) is the amplitude of the vibration of the tip.


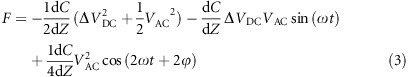


The KPFM feedback loop is used to adjust the *V*_bias_ to be equal to *V*_CPD_ and achieve Δ*V*_DC_=0. Thus, when the amplitude is zero and then the value of *V*_CPD_ is obtained. In our experiment, the value of *V*_CPD_ is the difference between the surface potential of the sample and the potential of the tip, as given by Equation (4):


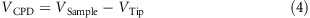


The relationship among the *V*_CPD_, the work functions of the sample (*φ*_Sampl*e*_) and the tip (*φ*_Tip_) can be written as^31, 32^,


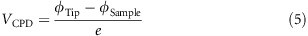


According to this relationship, a smaller *V*_CPD_ corresponds to a higher *φ*_Sample_. Thus, under UV illumination, the decrease of the surface potential around the Ag nanoparticle corresponds to an increase of the surface work function.

**Figure 2:**
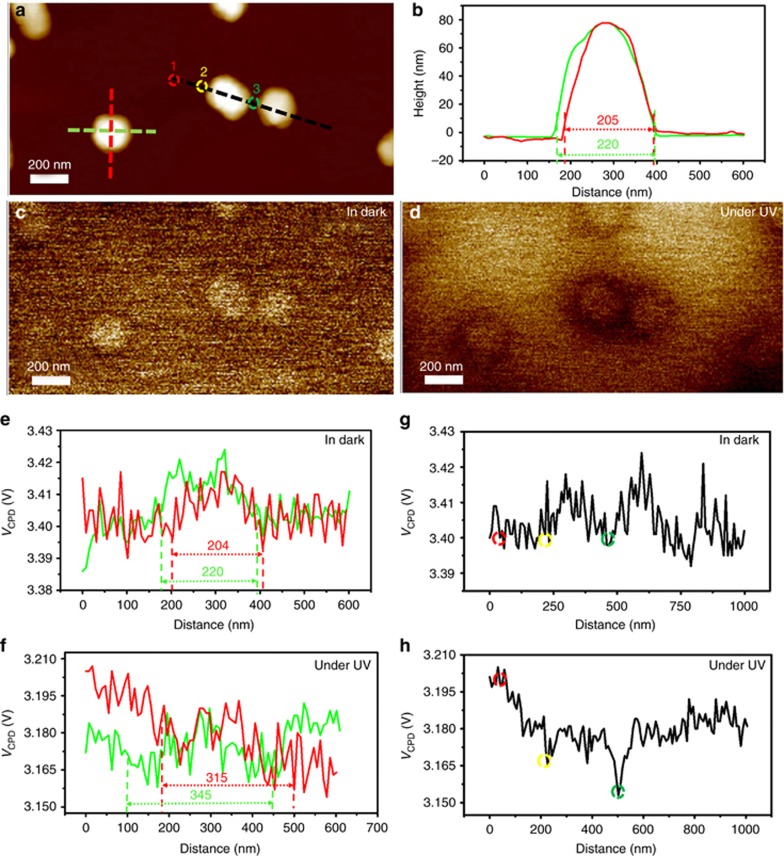
The characterization using AFM. (**a**) Surface morphology image of a Ag nanoparticle on GaN epilayer; (**b**) the section profile along the red and green lines crossing the isolated Ag nanoparticle in a surface morphology image. (**c**, **d**) are surface potential images obtained by KPFM in dark **c** and under UV (365 nm) illumination **d**. (**e**, **f**) are the section profile of surface potential obtained from **c** and **d**, respectively, along the red and green lines crossing one isolated Ag nanoparticle shown in **a**. (**g**, **h**) are the section profile of surface potential obtained from **c** and **d**, respectively, along the black line crossing dimer Ag nanoparticles shown in **a**.

The morphology of the Ag nanoparticles on the GaN epilayer was measured using AFM in tapping mode. The semi-spherical isolated Ag nanoparticles and dimer Ag nanoparticles are located randomly on the GaN epilayer, as shown in Supplementary Figs. S1 and S2. Figure 2a shows the typical morphology of the Ag nanoparticles on the GaN surface. The red and green lines are marked crossing the isolated Ag nanoparticle to describe the characteristics of the Ag nanoparticles clearly. The diameters of this Ag nanoparticle are 205 and 220 nm, corresponding to the red and green lines, respectively. The height of the Ag nanoparticle is ~80 nm, according to the section profiles of height given in Figure 2b. For the dimer of Ag nanoparticles, the black line crosses the two adjacent nanoparticles, as shown in Figure 2a. For the left and right nanoparticles of the dimer, the diameters are 278 and 229 nm, respectively, with corresponding heights of 70 and 60 nm, respectively.

The surface potential of a Ag nanoparticle on GaN was obtained by AFM using KPFM mode. Figure 2c and 2d, shows the measured surface potential in the dark and under UV illumination, respectively. Figure 2c indicates that under dark conditions, the surface potential of the isolated Ag nanoparticle was slightly higher than that of GaN. The section profiles of the surface potential along the same lines as the red and green lines are given in Figure 2e. In the dark, the surface potential of isolated Ag nanoparticle was ~3.415 V, which was higher than that of the GaN epilayer (3.400 V). The diameters of higher surface potential region were ~204 and 220 nm along the red and green lines, respectively, which were nearly equal to the size of the Ag nanoparticle. This result indicates that the surface potential in the dark was the intrinsic surface potential of the sample. Under UV illumination, the section profiles of the surface potential along the red and green line directions are shown in Figure 2f. The surface potential of the GaN epilayer next to the Ag nanoparticle (3.165 V) was lower than that of the GaN epilayer far from the nanoparticle (3.185 V). The diameter of the lower surface potential region was ~315 and 345 nm along the red and green lines, respectively. After subtracting the size of the Ag nanoparticle, the length of the surface potential reduction region was ~55 and 63 nm along the red and green directions, respectively.

A similar phenomenon was observed in the two adjacent Ag nanoparticles. For the dimer Ag nanoparticles on the GaN epilayer, three positions were marked with numbers 1–3 on the black line, as shown in Figure 2a. Position 1 was located far from the Ag nanoparticle, position 2 was in the vicinity of the Ag nanoparticle and position 3 was between the Ag nanoparticles of the dimer. In the dark, the surface potentials of positions 1, 2 and 3 are shown in Figure 2g; these surface potentials were found to be the same (3.400 V) as that of the isolated Ag nanoparticle in dark (Figure 2e). Under UV illumination, the surface potential decreased from 3.400 V to 3.200 V, 3.170 V and 3.150 V for positions 1, 2 and 3, respectively, as shown in Figure 2h. The light intensity was uniform at the three positions; thus, the dependence of the effects of photoillumination on the light intensity was not considered here. The reduction of the surface potential under UV illumination was observed for most of the Ag nanoparticles on the GaN epilayer, as shown in Supplementary Fig. S1.

As observed in the experiments, the distribution of the surface potential reduction at the GaN epilayer around a Ag nanoparticle was not uniform. For an isolated Ag nanoparticle, the closer it is to the boundary of Ag nanoparticle, the lower the surface potential is. For the dimer Ag nanoparticles, the surface potential (*P*) for positions 1–3 followed the sequence of *P*_1_>*P*_2_>*P*_3_. The contact between the Ag nanoparticles and the GaN surface should be a Schottky contact. However, the UV light cannot penetrate the Ag nanoparticle to generate a photocurrent; thus, no Schottky current was observed here. Even if there is a Schottky current of electrons from metal to semiconductor, it is normal to the surface of GaN and thus will not have an influence on the lateral decrease of the surface potential of GaN.

**Figure 3:**
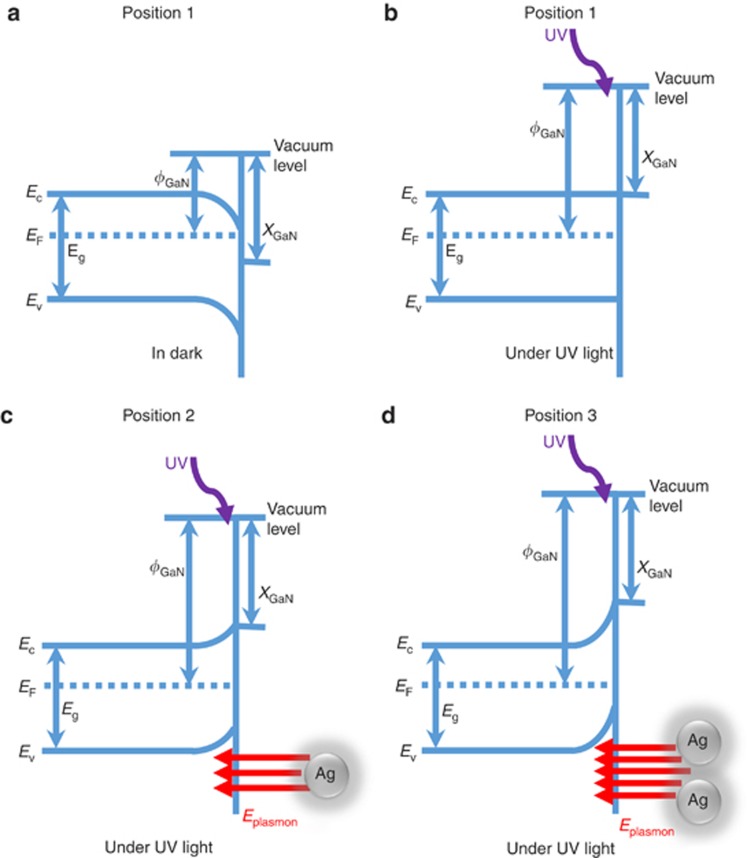
Schematic band diagrams of the GaN substrate at positions 1, 2 and 3 (positions 1–3 are shown in Figure 2a). The band structure of GaN under dark conditions with the donor surface state is shown in (**a**). For position 1, as shown in (**b**), under UV illumination, the photoelectrons move to fill all of the donor surface states; as a result, the band bending is recovered. For position 2, as shown in (**c**), the Ag SP induces an electric field around itself under UV light; as a result, the band bends upward and the electrons accumulate at the surface around the Ag nanoparticle, especially between the two nanoparticles, as shown in (**d**). Both the electric fields from the nanoparticles have an effect on it, resulting in intense band bending between the nanoparticles.

According to the principle of KPFM, the surface potential is related to the work function of the sample; thus, the band structure was analyzed schematically. The Schottky contact between the metal (Ag) and n-type GaN will lead to the pinning of the GaN conduction band (CB) to a constant level at the surface, resulting in the bending downward of the CB in the bulk. However, the energy band we show here is not underneath the Ag/GaN contact; rather, it is the energy band of the GaN in the vicinity of the GaN/Ag Schottky contact interface and the bulk GaN. Thus, the effect of the Ag/GaN Schottky contact on the surface potential can be ignored. Here we take the typical dimer Ag nanoparticles as an example. In the dark, the surface potential at positions 1–3 has the same value of ~3.400 V. Under UV illumination, all of the surface potentials at the three positions decreased. For position 1, which was far from the boundary of the Ag nanoparticle, the decrease of the surface potential originated from the surface state of the GaN epilayer. There are empty donor surface states in the GaN epilayer that lead to the downward bending (*qV*_D_) at the surface of the GaN epilayer^33, 34^, as shown in Figure 3a. Under UV illuminates, the photogenerated electrons move to the surface states until the surface states become occupied with electrons and the band bending recovers to the flat band condition (as shown in Figure 3b). As a result, the surface work function increased and the surface potential decreased from 3.4 to 3.2 V at position 1.

At position 2, in the vicinity of a Ag nanoparticle, the surface potential of GaN decreased from 3.400 V before UV illumination to 3.170 V and after UV illumination; that is, in addition to the electrons filling the surface states, more electrons accumulated at position 2. These excess electrons caused the band to bend upward with the value ~30 mV, as shown in Figure 3c. At position 3, more electrons were accumulated than at position 2. The surface work function increased further, and the surface potential decreased further, as observed in Figure 2d. The surface potential of GaN decreased from 3.400 to 3.150 V. The band bent upward with the value ~50 mV.

The only possible reason for the ultra-accumulation of electrons at positions 2 and 3 is the SP-induced localized field enhancement by Ag nanoparticles. Under UV illumination, the SP effect results in localized field enhancement. Next, the plasmon-enhanced field drives the accumulation of photogenerated electrons at the positions of the GaN epilayer in the vicinity of the Ag nanoparticles, leading to the increase of the surface work function, the upward bending of the energy band and, thus, the reduction of the surface potential of the GaN epilayer. For the dimer Ag nanoparticles, because of the interaction between the particles, the electric field at the gap is higher than that of at other positions. It can also be understood as follows: the electric fields generated by the two nanoparticles will be overlapped at the gap between the nanoparticles, resulting in a higher electric field at position 3. More electrons are accumulated at this position, resulting in a higher surface work function and lower surface potential.

Supplementary Fig. S1 also shows that the decrease of the surface potential depends on the size of the Ag nanoparticles. Under UV illumination, obvious surface potential reduction was observed in the vicinity of the Ag nanoparticles with sizes in the range of 140–280 nm (according to the statistical analysis of Supplementary Information S3), which again proved that the reduction of the surface potential around a Ag nanoparticle originates from the SP-localized field enhancement.

**Figure 4:**
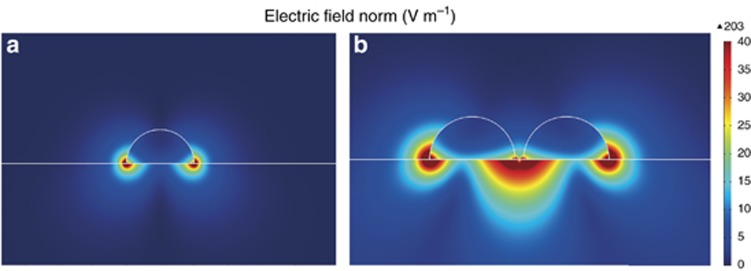
The SP electric field distribution of an isolated Ag nanoparticle and a dimer of Ag nanoparticles.

To further understand the analysis above, the surface spatial distributions of the electric field for Ag nanoparticles on GaN was also simulated using time-domain and frequency-domain finite-element methods. We simulated the isolated and dimer Ag nanoparticles with diameter of 200 nm with the gap of 30 nm. The incident wavelength was *λ*=350 nm. Figure 4a and 4b, shows the electric field distribution of semi-spherical Ag nanoparticles on the GaN epilayer. Under UV illumination, localized field enhancement was observed to be generated in both isolated and dimer Ag nanoparticles. For the dimer Ag nanoparticles, the interparticle coupling interaction between the particles causes the electric field to be the highest.

Thus, our observation verifies that the cause of the enhanced responsivity of the GaN UV detector via Ag nanoparticles was due to the localized field enhancement effect by SP. Theoretically, the enhancement factor depends strongly on the size and the surrounding dielectric environment of the particles. Based on SP theory, if the nanoparticle sizes are less than 20 nm, then most of the energy of the SP collective modes is transferred to electronic transitions to induce hot carriers, whose distribution is quite limited in a narrow phase space around the Fermi level. However, if the nanoparticle sizes are >20 nm, then most of the SPs’ energy is radiative, and photons are emitted^35^.

In such a case, the SP field can interact directly with the interband electronic transitions in dielectric materials when Ag nanoparticles are attached to the GaN surface. The interaction can be dependent on the plasmon’s polarization and result in localized field enhancement. To understand the observed experimental results, we theoretically calculated the enhancement in the polarizability for a spheroid with a>*b*, where *a* and *b* are semi-major and semi-minor axes of the spheroid, respectively. As shown in Figure 4, our FDTD simulation, using the Comsol program, utilizes a numerical method to solve Maxwell equations. These simulations can provide the electric field distribution formed by the Ag nanoparticles but fail to provide us with the physical insight that is required to understand the response of the metal particles to light. For the size of the nanogeometry considered in our experiment, a full analytical solution is difficult to achieve. Nevertheless, for nanogeometries of sizes ~150 nm or higher, previous authors^36^ developed an analytical solution for the polarizability for a sphere and spheroid that are in complete agreement with the experimental results. These simple and elegant solutions can be derived by an expansion of the first TM mode of Mie’s formulation. The retardation effect is explicitly considered.

**Figure 5:**
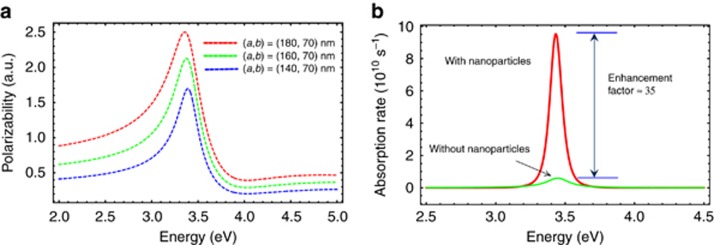
(**a**) The polarizability for a nanospheroid of different sizes, as given by the numbers in the figures using the analytical expression given in Ref. 39. (**b**) The enhancement in the absorption rate for a spherical nanoparticle using the quasi-static approximation. In both cases, the dielectric environment is taken to be a vacuum.

We implement the analytical expression developed in Ref. 36 that also accounts for the effect that arises due to a finite speed of light for a spheroid with an eccentricity, 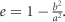 In Figure 5a, we plot the polarizability as function of energy for different sizes of nanospheroids with a vacuum dielectric environment. The polarizability is enhanced at ∼ 3.400 eV, indicating that surface plasmons are excited on the metal surface and thus contribute to the enhancement of the responsivity obtained in our previous experiment^18^.

It is useful to analytically estimate the enhancement in response occurring around the GaN bandgap energy for smaller-sized nanoparticles by using the quasi-static approximation. This approximation works well for small-sized nanoparticles and provides an estimate for larger-size nanoparticles if the dielectric environment is less dispersive (*ε* is close to 1)^37^. The interaction between the plasmon field and the electronic system in the bulk can be described by an interaction Hamiltonian^38^,


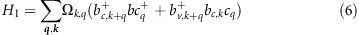


where *b*_*c*_(*b*_*v*_) is the electron annihilation operator in conduction (valence) band and *c_q_* is the plasmon annihilation operator. 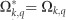 is the Rabi frequency for the interaction between plasmon of wave vector *q* and the direct electronic transition with wave vector *k* and is given by


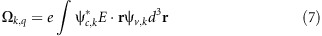


where *e* is the electronic charge, ψ*_c_* (ψ*_v_*) is the conduction (valence) band wave function, ***E*** is the plasmon field at the dielectric materials and **r** is the dipole radius vector. Equation (7) shows that the plasmon field interacts directly with the electronic transition in the bulk. In nanoparticles of size where surface scattering of electrons contributes significantly in plasmonic loss, a single quantum of plasmon decays in to a ‘hot’ electron–hole pair due to initial and final momentum difference of electron is contributed by the size effect. However, for larger-sized nanoparticles, such an effect is strongly suppressed. The hot spot around the nanoparticles on the surface can generate a strong electric field, which can induce a transition in GaN. The electronic transition probability per unit time induced by the plasmon field can be calculated using Fermi’s golden rule and is given by,


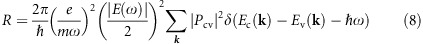


where *P*_cv_ is the momentum matrix element in GaN and *m* is the free electron mass. The delta function in Equation (8) ensures the momentum conservation without phonons. It can be easily shown that 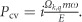 Assuming that the fields do not penetrate and reach the substrate, the static plasmon field established on the surface of nanoparticle can be approximated by the quasi-static approximation as 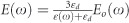, where *ε*_*d*_ and *ε*(*ω*) are the dielectric for the medium and the metal, respectively, and *E*_*o*_(*ω*) is the electric field of incoming photons. The dominant transition occurs at approximately the gamma point of the Brillouin zone of GaN, the momentum matrix element for which is 8.85 × 10^−20^ in cgs units^39^. A relevant quantity is a Poynting vector, *S*_P_, which describes the power flow across an arbitrary cross-sectional area and is given by 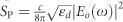. In Figure 5b, we show the absorption per unit time using a plasmon field on the dielectric surface. The absorption rate is proportional to the responsivity (that is, the absorption rate is also equivalent to the photocurrent). The calculation shows that because of the localized field enhancement effect by SP, the enhancement can be up to 35 times with nanoparticles relative to the photocurrent of the device without nanoparticles. The photocurrent enhancement of a single nanoparticle is equivalent to the average surface current per unit area of the aggregate nanoparticles. Note that the validity of the quasi-static approximation is limited to a regime in which the dipole radius vector of the electro-mechanical oscillations (plasmon oscillation) that is covered in one optical cycle is comparable to the size of the nanoparticle (negligible retardation effect).

## Conclusions

Here we observed a reduction in the surface potential in the vicinity of Ag nanoparticles by using KPFM. Under UV illumination, for an isolated Ag nanoparticle of 200 nm in diameter, the surface potential reduction region was within ~60 nm of the boundary of the Ag nanoparticle. For a dimer of Ag nanoparticles, the lowest surface potential was observed into the gap between the Ag nanoparticles. We verified the SP-induced localized field enhancement by Ag nanoparticles on the GaN epilayer by using the undamaged method of KPFM. Based on the measured SP effect, the physical mechanism for the enhanced UV responsivity of GaN detectors using Ag nanoparticles was also discussed theoretically. The results presented here not only provide direct experimental proof to verify the localized field enhancement and allowed us to understand the SP effect further but may also accelerate the deployment of semiconductor optoelectronics devices using SP enhancement.
