# Supplementary material for: Direct observation of localized surface plasmon field enhancement by Kelvin probe force microscopy
Source: Light Sci Appl. 2017 Aug 25;6(8):e17038–. doi: 10.1038/lsa.2017.38 (PMC6062315; doi:10.1038/lsa.2017.38)
Supplement: Supplementary Information [file lsa201738x1.docx]

**Supplementary Information for**

**Direct Observation of Localized Surface Plasmon Field Enhancement by Kelvin Probe Force Microscopy**

*Dabing Li^1^*, Xiaojuan Sun^1^*, Yuping Jia^1^, Mark I Stockman^2^, Hari P. Paudel^2^, Hang Song^1^, Hong Jiang^1^, Zhiming Li^1^*

Prof. Dabing Li, Dr. Xiaojuan Sun, Dr. Yuping Jia, Prof. Hang Song, Prof. Hong Jiang

State Key Laboratory of Luminescence and Applications, Changchun Institute of Optics, Fine Mechanics and Physics, Chinese Academy of Sciences, Changchun 130033, P. R. China

Email: lidb@ciomp.ac.cn, sunxj@ciomp.ac.cn

Dr. Hari P. Paudel, Dr. Mark I. Stockman

Center for Nano-Optics (CeNO) and Department of Physics and Astronomy,

Georgia State University, Atlanta, Georgia 30340, USA

Correspond author information is as follows:

State Key Laboratory of Luminescence and Applications,

Changchun Institute of Optics, Fine Mechanics and Physics, Chinese Academy of Sciences, 3888 Dong Nan Hu Road, Changchun 130033, P. R. China

Phone and fax: +86-431-86708157

Email: lidb@ciomp.ac.cn; sunxj@ciomp.ac.cn

**S1: Characterizations of Ag nanoparticles on GaN substrate by AFM and KPFM.**

The morphology of Ag nanoparticles on GaN substrate were determined using atomic force microscopy (AFM) at three areas as shown in Fig.S1a, S1c and S1e. Meanwhile, the correspondence surface potential was determined using Kelvin Probe force microscopy (KPFM) as shown in Fig.S1b, S1d and S1f. Under ultraviolet illumination, the potential of GaN substrate around Ag nanoparticle was lower than other regions commonly. This phenomenon was the result of the plasmonic enhancement as analysis in the main text. However, the Ag nanoparticles numbered with green number has no plasmonic enhancement. The Ag nanoparticles marked with black number has prominent enhancement efficiency.


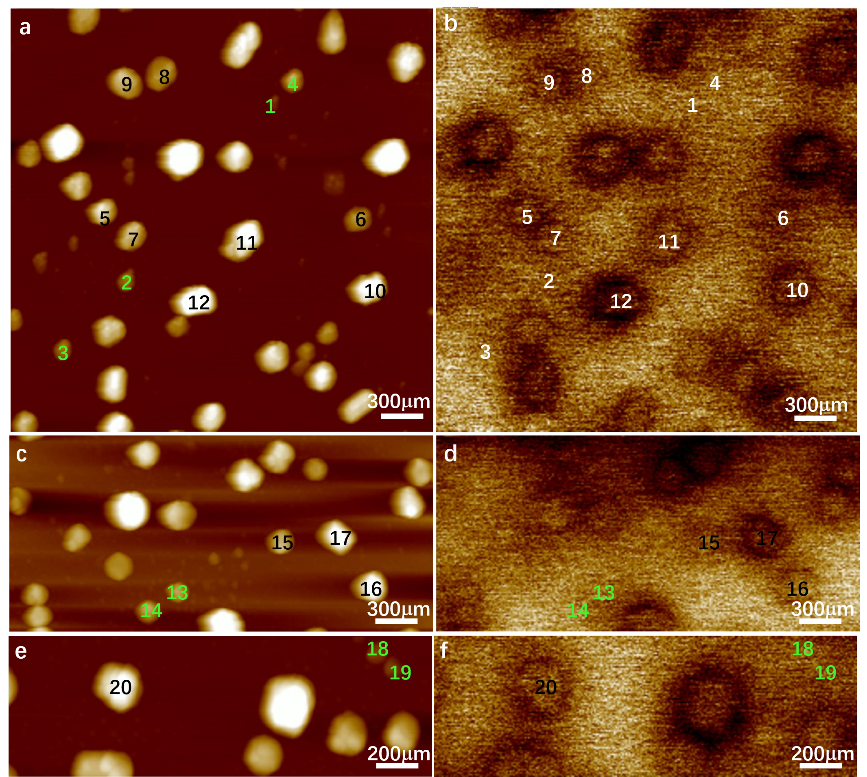


Fig.S1 The AFM morphology images of Ag nanoparticles on GaN substrate in **a**), (**c**) and (**e**). The correspondence KPFM surface potential images in (**b**), (**d**) and (**f**).





**a**





**b**

Fig. S2 SEM images of Ag nanoparticles.

**S3: Statistical data of Ag nanoparticles with nanoplasmonic enhancement.**

The statistical data of Ag nanoparticles which has nanoplasmonic enhancement is shown in the Table S1. Here the isolate Ag nanoparticles are chosen, and the number of them is given which is according with the number marked in Fig.S1. In the table, the size of nanoparticles is collected and so does the ΔV_CPD_ and the size of the electric field. Here ΔV_CPD_ is the difference of V_CPD_ between the GaN around and far away from Ag nanoparticles. The larger ΔV_CPD_ means more electrons accumulating at this area. The electric field size is used to descript the low surface potential area around Ag nanoparticles, which shows us the effective region of the electric field caused by the Ag nanoparticles SP effect.

Table S1 The properties of Ag nanoparticles with nanoplasmonic enhancement.

| **number** | **ΔV_CPD_/mV** | **Nanoparticle size/nm** | **electric field size/nm** |
| --- | --- | --- | --- |
| **16** | **19.5** | **218** | **64.75** |
| **20** | **25** | **234** | **42** |
| **10** | **18** | **241** | **85.5** |
| **17** | **19.7** | **247** | **50** |
| **12** | **35.6** | **270.5** | **97** |
| **11** | **8** | **282.5** | **76.25** |
